# Dynamic Morphological Computation Through Damping Design of Soft Continuum Robots

**DOI:** 10.3389/frobt.2019.00023

**Published:** 2019-04-11

**Authors:** Antonio Di Lallo, Manuel G. Catalano, Manolo Garabini, Giorgio Grioli, Marco Gabiccini, Antonio Bicchi

**Affiliations:** ^1^Dipartimento di Ingegneria dell'Informazione, Centro di Ricerca E. Piaggio, Università di Pisa, Pisa, Italy; ^2^Dipartimento di Ingegneria Civile e Industriale, Università di Pisa, Pisa, Italy; ^3^Fondazione Istituto Italiano di Tecnologia, Genoa, Italy

**Keywords:** soft robotics, damping, under-actuation, dynamic response, soft material robots, soft continuum robots

## Abstract

Inspired by nature, soft robotics aims at enhancing robots capabilities through the use of soft materials. This article presents the study of soft continuum robots which can change their dynamic behavior thanks to a proper design of their damping properties. It enables an under-actuated dynamic strategy to control multi-chamber pneumatic systems using a reduced number of feeding lines. The present work starts from the conceptual investigation of a way to tune the damping properties of soft continuum robots, and leverages on the introduction of viscous fluid within the soft chamber wall to produce dissipative actions. Several solutions are analyzed in simulations and the most promising one is tested experimentally. The proposed approach employs a layer of granular material immersed in viscous silicone oil to increase the damping effect. After validation and experimental characterization, the method is employed to build soft continuum actuators with different deformation patterns, i.e., extending, contracting and bending. Experimental results show the dynamic behavior of the presented actuators. Finally, the work reports information on how the actuators are designed and builded, together with a discussion about possible applications and uses.

## 1. Introduction

In recent years soft systems are raising more and more interest in the robotic community and an increasing number of applications have been developed (Tan et al., 2007; Rus and Tolley, 2015). Two main approaches are adopted to build robots with compliant behavior (Della Santina et al., 2017): the first takes inspiration from the vertebrate musculoskeletal system and relies on flexible joints, achieved especially by means of variable impedance actuators (soft articulated robots) (Vanderborght et al., 2013; Yu et al., 2015), while the other derives its concept from invertebrates and employs elastomeric materials to make continuosly deformable structures (soft continuum robots) (Cowan and Walker, 2008). The high activity in this research topic led to the conception of multi-material fabrication processes and integrated design principles, involving new soft actuators and sensors.

Indeed, softness in biological structures is a key factor in interacting with the environment and in responding to external and internal forces (Kim et al., 2013). In 2006 the concept of *morphological computation* was introduced to explain the role of softness in a mechanical system (Pfeifer et al., 2006). Indeed, *morphological computation* indicates that the role of the body mechanics is of fundamental importance in how a body can interact and adapt its shape to the environment: this can enormously facilitate—or hinder, if done in the wrong way—the control action. A key element to achieve such intelligent behavior is the softness of the robot body. As a consequence, one of the materials that are most widely employed to build soft continuum robots is silicone.

From the actuation point of view, several schemes are used to operate soft robots, including tendons (Calisti et al., 2011), shape memory alloys (Laschi et al., 2012), muscle-like actuators (Chou and Hannaford, 1996), just to cite a few. For more details, the interested reader can refer to the dedicated review article (Boyraz et al., 2018). A great range of applications capitalize on the consolidated background on electrical components and of recent progresses in electroactive polymers (EAPs) and in additive manufacturing technologies to design electrically powered soft actuators and sensors with increased capabilities (Rossiter et al., 2009). Despite such progress, the need for reducing the number of rigid components has given a renewed pulse to fluidic actuation, with a particular consideration for pneumatics (Wehner et al., 2014). Some untethered mobile robots rely on the direct generation of the working pneumatic pressure from a chemical reaction for the power supply (Onal et al., 2017). The same approach has been exploited in Wehner et al. (2016), where the authors created a complete micro-fluidic circuit replicating its analogous electronic scheme. Soft robotics also opened challenges concerning the modeling and the control techniques to rule largely deformable systems, due to their non-linear response. A common strategy involves the analysis and description of such structures through experimentally validated, quasi-static analytical and finite-element models. In this approach, a relevant role is played by the fiber reinforcement: by varying the winding angle of fibers a range of different deformation behaviors can be achieved (Connolly et al., 2015; Polygerinos et al., 2015b). Moreover, the combination of several actuators allows to obtain complex motions with multiple degrees of freedom (Connolly et al., 2015).

However, one of the disadvantages of classical pneumatic control lies in its bulkiness and heaviness. In fact, the actuation system usually includes a pressure generator, a pressure regulator and at least one valve for each chamber, with its relative feedline. This limits the overall system performance especially for mobile and wearable applications. In literature several approaches have been proposed with the purpose to simplify the actuation architecture (e.g., in Booth et al., 2018), the authors propose a miniature pneumatic regulator for direct integration into compact soft robots which allows to simplify valve control in such systems. Another path explores the use of passive valves activated through a specific modulation of the input pressure (Napp et al., 2014). Pneumatic networks of small channels in elastomeric materials are appealing for producing sophisticated motions with a reduced number of controls. In Mosadegh et al. (2014), the authors focus on the rate of actuation of pneu-nets and describe a new design that bends rapidly. Furthermore, it is shown that by changing the rate of pressurization, two different types of motion can be obtained from the same prototype. We already introduced the importance of a rate-dependent behavior in our previous work (Piazza et al., 2016; Di Lallo et al., 2018), where we presented a method to implement a simple yet effective dynamic morphological computation strategy to soft articulated robots. That method is based on the co-design of the mechanical parameters of the system modules and of the input signal, with particular attention to the design of the damping components. So far, its application to soft robots involved only systems where compliance is concentrated in some parts, and these are connected to discrete and rigid components.

In this work the authors want to bring such kind of concept in the context of soft continuum robots, in which instead the compliant behavior is distributed throughout the structure. The challenges to address this goal are many, but among them the problem of independently designing the stiffness and damping properties of elastomeric chambers in a distributed manner prevails. To overcome this problem is fundamental to enable the realization of integrated soft continuum systems with true dynamical *morphological computation* abilities. This crucial step looks at how to change the dynamical behavior of soft silicone chambers, in order to implement different typologies of, so called, *dynamic soft actuators*. We move toward this goal investigating an approach that relies on the introduction of fluids within the chamber walls to change their damping behavior. Different implementations are conceived, presenting the intuition behind the proposed designs, and then are preliminary compared in simulation. The most promising approach, based on the introduction of granular material in the viscous oil is tested experimentally, and then used to propose the fabrication of three different typologies of soft continuum actuators with different damping behaviors, designed to actuate motions of extension, contraction and bending.

This paper is articulated as follows: in section 2 the general problem involving conventional multi-chamber fluidic systems is discussed together with our proposed solution, based on an under-actuated control strategy. The rationale behind the application of the envisioned method to soft continuum robots is presented and the related challenges are described. In section 3 a possible approach is investigated with a theoretical insight first, and through preliminary Finite Element simulations after. The most promising approach is here experimentally validated and characterized. Consequently, the three soft actuators are presented and discussed in section 4, together with the description of the fabrication process adopted to fabricate them. Section 5 reports the results of a validation campaign conducted to investigate the dynamical behavior of the actuators. Finally in section 6 we show and discuss some application examples and in section 7 we draws conclusions and traces the route for future work.

## 2. Background and Problem Definition

Usually, soft fluidic systems consist of multiple independent chambers individually controlled through a complex network of pipes and active valves. This allows to easily obtain a large variety of movements, which is a desirable property for several applications. Some examples include: hands, where the order of the fingers closing can yield different grasping patterns (see e.g., Deimel and Brock, 2013; Piazza et al., 2016); worm-like robots, as in Shepherd et al. (2011) and Tolley et al. (2014), or snake-like robots, as in Greer et al. (2018), which could adapt their gait in order to move across different terrains or to navigate difficult obstacles; anymaloids as the soft robotic fish presented in Katzschmann et al. (2018), where the soft material is used to replicate the soft behavior of a fish fin, or the soft octopus robot presented in Laschi et al. (2012); manipulators as in Lee et al. (2016) or Takeichi et al. (2017), where the Giacometti continuum soft arm is used for industrial inspection; and medical devices, as in (Roche et al., 2017) where a soft robotic sleeve is used to assist cardiovascular function in an *in vivo* surgery, or as in Polygerinos et al. (2015a) where a soft hand exoskeleton is made with continuously deformable parts.

**Figure 1:**
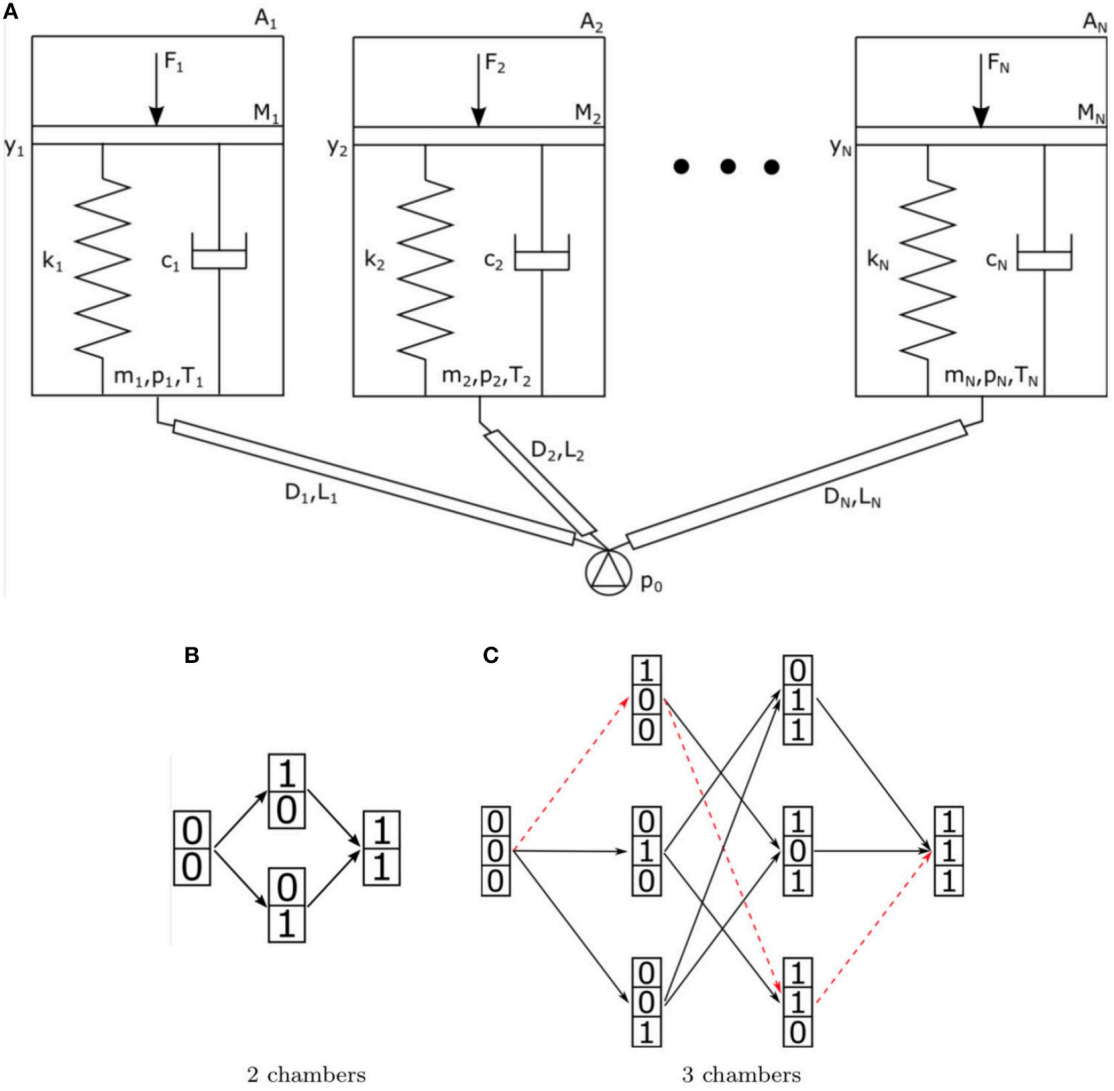
**(A)** Schematic representation of a fluidic network with multiple chambers connected in parallel to the same input source. Each chamber is modeled as a mass-spring-damper system. **(B,C)** Possible states configurations and behaviors for two systems. Column vectors of 0 and 1 s correspond to system deflation/inflation states, arrows correspond to different inflation actions. **(B)** corresponds to a 2-chamber system, while **(C)** to a 3-chamber one. A particular behavior is highlighted by the red dashed line.

All these systems implement different functions mostly thanks to the possibility of inflating/deflating their chambers in different sequences. The abundance of control inputs allows their control with very good performances, but this comes at the cost of making the system heavy and bulky. Our aim is the simplification of the pipe and valve robot actuation network to obtain different behaviors in the robot, with a reduced set of independent control inputs. Our base hypothesis is that different behaviors can be implemented by eliciting different inflation/deflation sequences in the different chambers. Model the robot with a pneumatic network composed of *N*-inflatable chambers, connected in parallel to the pressure source, as shown in Figure 1A. Each chamber is modeled as a piston with a finite stroke, coupled to a spring and a damper, simulating both the equivalent mechanical properties of the chamber and of the connected robot structure. Assuming air as an ideal gas and flow laminar, we can write the dynamics of the system governed by the equations of motion of the pistons and by the mass and energy balances of the air flow. Each chamber can be described through the following system of nonlinear differential equations (White, 1999):

(1, 2, 3 and 4) $$\begin{array}{l} \{\begin{array}{ll} {\dot{m}}_{i} & =\frac{p_{0}-p_{i}}{Z_{i}}\\ {\ddot{y}}_{i} & =\frac{1}{M_{i}}\left(\left(p_{i}-p_{\text{atm}})A_{i}-F_{i}-k_{i}(y_{i}-l_{\text{r}i})-c_{i}{\dot{y}}_{i}\right)\right)\\ {\dot{p}}_{i} & =\frac{R}{c_{v}A_{i}y_{i}}\left({\dot{m}}_{i}c_{p}T_{\text{atm}}-\frac{c_{p}}{R}A_{i}p_{i}{\dot{y}}_{i}+k_{\text{w}}S_{i}(T_{i}-T_{\text{atm}})\right)\\ T_{i} & =\frac{p_{i}A_{i}y_{i}}{m_{i}R} \end{array} \end{array}$$

where

(5) $$\begin{array}{l} Z_{i}=\frac{32\mu L_{i}}{\pi \rho_{i}D_{i}^{4}} \end{array}$$

(6) $$\begin{array}{l} \rho_{i}=\frac{m_{i}}{A_{i}y_{i}} \end{array}$$

(7) $$\begin{array}{l} S_{i}=y_{i}\sqrt{4\pi A_{i}} \end{array}$$

The definition of symbols follow: μ, dynamic viscosity of the air, *p* absolute pressure, *p*_0_ supply pressure, *p*_atm_ atmospheric pressure, *R* specific gas constant, ρ density of the air, *S* external surface of the piston, *T* temperature, *T*_atm_ atmospheric temperature, *y* piston height, *A* piston area, *c* damping coefficient, *c*_*p*_ heat capacity at constant pressure, *c*_*v*_ heat capacity at constant volume, *D* diameter of the duct, *F* external force, *k* stiffness, *k*_w_ convective heat transfer coefficient, *L* length of the duct, *l*_r_ rest length of the spring, *m* mass of the air in the piston, *M* mass of the piston, *Z* ratio between pressure drops and flow rate, and the subscript *i* refers to the generic chamber.

Given a chamber *a*, we describe its state as fully inflated (*a* = 1) or deflated (*a* = 0). Consequently, the state of the global system can be described by a vector of *N* binary digits. We define as *behavior* each possible sequence of inflation (or deflation) of the different chambers. Figures 1B,C illustrates e.g., the sets of all inflation behaviors for two systems with two and three chambers, respectively. Each oriented path, from the leftmost state to the rightmost state, along the arrows indicates a possible inflation behavior (simultaneous inflations are neglected for brevity). Example of Figure 1B has two possible behaviors, while the one of Figure 1C, with three chambers, has six. Our design objective, stated in Di Lallo et al. (2018), is, given a subset of *n* possible behaviors, to determine the control input *p*(*t*) and the design of the mechanical parameters of the system, such that all the *n* behaviors can be achieved.

**Figure 2:**
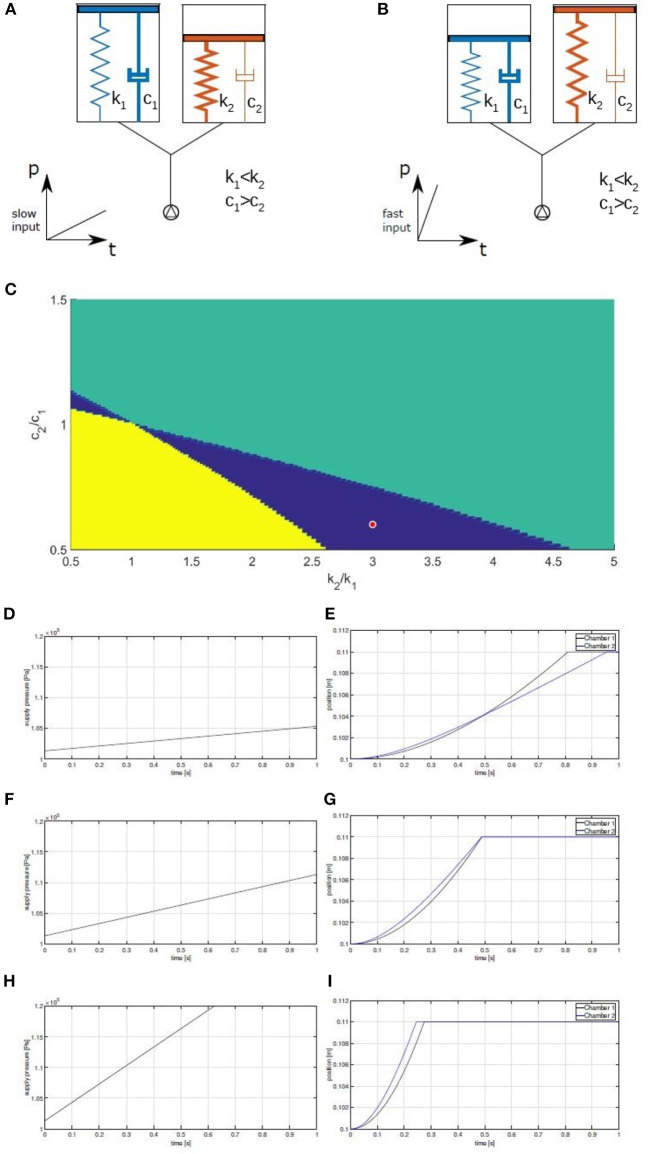
A fluidic network, subjected to two different inputs. The right chamber has higher stiffness than left chamber, while left chamber has higher damping than the right one (i.e., the reverse). For slow inputs **(A)** the chamber on the right inflates first, while for faster inputs **(B)** the one on the left is faster. **(C)** Behaviors of two chambers, as a function of the ratios of stiffness *k*_2_/*k*_1_ and damping *c*_2_/*c*_1_. Regions in blue indicate that different inputs produce different behaviors in terms of order of inflation. Yellow or green areas denote the regions where chamber 1 or 2, respectively, always inflates first. Blue area is our design space. **(D–I)** Evolution of positions with respect to different supply pressure gradients.

To practically describe the concept we can take in consideration a system composed of two inflatable chambers only, connected in parallel to the same pressure source. Each chamber is modeled as a piston with a finite stroke, coupled to a spring and a damper, simulating both the equivalent mechanical properties of the chamber and of the connected robot structure. As discussed in Di Lallo et al. (2018), a proper co-design of mechanical impedance properties allows to leverage on the actuation rate in order to elicit different behaviors of the overall system. The key idea is that by playing on the speed of inflation it is possible to render the dynamic response of the damper dominant over the effect of the spring or vice-versa. When pressure grows quickly damping plays the greatest role, while at low pressure gradients stiffness dominates. A sketch of this idea is shown in Figures 2A,B.

Assume that the stiffness and the damping of the two chambers can be designed freely. The goal of the task is to determine the mechanical parameters of the two chambers and two pressure profiles such that both inflation sequences are possible. By simulating the system, it is possible to identify the values of the mechanical parameters for which the intended behavior manifests. Figure 2C shows the results of such a simulation campaign, highlighting the set of mechanical parameters that satisfy our specifications.

As an example, Figures 2D–I show the behavior of the system corresponding to the red dot in Figure 2C when three different pressure profiles are applied. Chamber 2 is 0.6 times less damped and 3 times stiffer than chamber 1. No external forces are acting on the chambers, except for the end-stroke limits. It is possible to notice how the steepest pressure profile lets chamber 1 inflate before chamber 2, while the middle pressure profile lets the two chambers inflate at the same time. Finally, the slowest pressure ramp lets chamber 2 inflate before chamber 1. Figure 2 highlights also one possible drawback of the proposed technique: the duration of the inflation itself can not be made independent from the desired behavior. While this is an important aspect to keep in mind when applying this design method, it doesn't represent a major problem in non-time-critic applications.

While in a lumped system this behavior can be easily obtained through an opportune design of springs and dampers (as shown in Di Lallo et al., 2018), it is cumbersome to obtain when dealing with soft continuum systems.

Therefore, such an approach opens new challenges concerning the independent tuning of the mechanical parameters, i.e., stiffness and damping, of a single soft silicone chamber. In principle this can be obtained via a proper selection of the intrinsic properties of the material (e.g., elastic modulus, viscosity) and a suitable choice of geometrical parameters (e.g., thickness of the chamber wall). In the following a brief discussion presents the advantages and drawbacks of this approach when applied to soft continuum robots. If, on one side, it results quite easy to modify the stiffness of a soft chamber by acting on the thickness of the chamber wall or on the elastic modulus of the material (silicone in our case), it is much more difficult to change the damping behavior of a soft material, especially if large changes are needed, and if we want to maintain independent design of stiffness and damping (Fincan, 2015). Attempts have been made working from a chemical perspective, i.e., by modifying the material composition through additives, but there is not yet a general rule for what still remains substantially an open research area (Wu and Akiyama, 2002; Galantini et al., 2013). Therefore, every modification requires a new characterization of the material. Various polymers with additives, that combine the characteristics of elastic solids and Newtonian fluids, have been demonstrated. The characterization exploits a Dynamic Mechanical Analysis to study the visco-elastic properties of the material (Menard, 2008) (via stress-strain tests at varying temperature and frequency) which implies a great cost in terms of time. Moreover, the change of the chemical composition of the materials has the inconvenience of affecting simultaneously the elastic and the viscous properties, making very difficult to achieve a certain damping ratio, with a given stiffness. Hence, this work investigates an approach that relies on the introduction of viscous fluids in the chamber wall to achieve the desired damping. This approach leads us to the possibility of dividing the design problem of the chamber wall into two sub-problems where damping and stiffness are the major requirements. In the next section we devise how to successfully integrate damping components in silicone chambers.

## 3. Proposed Solution

A classical solution adopted in mechanics to generate damping actions resorts to dissipating forces produced in a viscous fluid. Where common mono-dimensional dampers do not represent a viable approach, alternative solutions have been conceived in literature in order to adapt the same principle to specific situations. As an example, a method for providing damping to a bearing assembly of a gas turbine engine is described in Ertas et al. (2017). It is based on the flow of a damper fluid through a restrictive channel that puts in communication two cavities with semi-rigid walls. Other applications to deformable systems include fluid dampers with a flexible damping plate (Jones, 2000), porous elastic sheet dampers with a magnetic fluid (Liu, 2009), and several designs of fluid-and-elastomer dampers (Schmidt and McGuire, 1994; McGuire, 1996, 2000), which exploit the combination of viscous and hysteretic damping for vibration suppression. The viscous damping effect is generated if tangential sliding is present between parcels of the fluid, hence the choice of the location of the fluid inside the chamber wall is critical.

**Figure 3:**
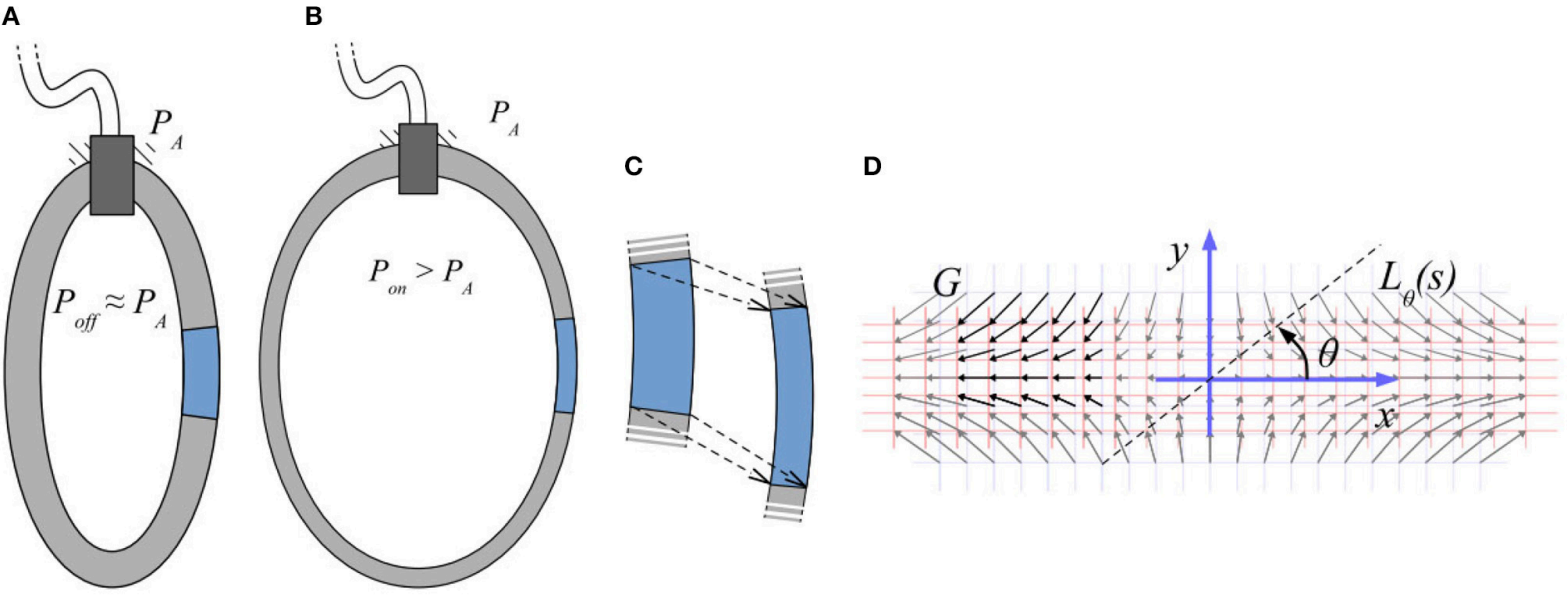
Scheme of an inflatable chamber in rest conditions **(A)** and after pressurization **(B)**. Deformation of a macroscopic element of the chamber wall **(C)**. Deformation map for a 2D rectangular model **(D)**.

Considering one finite element of the surface membrane, the main deformation it undergoes when the chamber is subject to inflation (see Figures 3A–C), can be described by the combined effect of two actions: a stretching action, due to the fact that the inflating chamber will present a larger external surface, and a compression action, due to the effect of the different pressures acting on the two walls of the membrane. We can attach a coordinate map (see Figure 3D for variable definitions) to the element and look at the local deformation speed induced by the aforementioned macroscopic deformation.

Define *P*(*t*) the trajectory of a generic point in function of time. Assuming the initial conditions for *t* = 0 are $P\left(0\right)={\left[x_{0},y_{0}\right]}^{T}$, and assuming, for simplicity, an isotropic transformation which evolves linearly over time, the deformed position at generic time *t* can be defined as

(8) $$\begin{array}{l} P\left(t\right)=\left[x\left(t\right),y\left(t\right)\right]={\left[x_{0}+\alpha x_{0}t,y_{0}-\nu \alpha y_{0}t\right]}^{T}, \end{array}$$

where α is a geometric constant, indicative of the magnitude of the deformation process, and ν is a second geometric constant describing the ratio of deformation speed in the two directions normal and tangential (analogous to the Poisson ratio). The local speed of deformation field can be calculated as

(9) $$\begin{array}{l} G≜\frac{dP}{dt}={\left[\alpha x_{0},-\nu \alpha y_{0}\right]}^{T}. \end{array}$$

If we now chose a direction of observation of this vector field, defined by a line passing by the origin of our reference frame at an angle θ, see Figure 3D given by the parametric equation $L_{\theta }\left(s\right)={\left[s\cos\left(\theta \right),s\sin\left(\theta \right)\right]}^{T}$, we can define the component of *G* that is orthogonal to the line *L*_θ_(*s*), as

(10) $$\begin{array}{l} G_{⊥\theta }=\left[-\sin\left(\theta \right),\cos\left(\theta \right)\right]{\left[\alpha s\cos\left(\theta \right),-\nu \alpha s\sin\left(\theta \right)\right]}^{T}\\ =-\frac{1}{2}\left(1+\nu \right)\alpha s\sin\left(2\theta \right). \end{array}$$

Finally, we can compute the geometric shear strain rate $\dot{\gamma }$ as the derivative of the previous with respect to *s*, as

(11) $$\begin{array}{l} \dot{\gamma }≜\frac{\partial G_{⊥\theta }}{\partial s}=-\frac{1}{2}\left(1+\nu \right)\alpha \sin\left(2\theta \right). \end{array}$$

As Equation (11) suggests, even a simple rectangular chamber of material, isotropically deforming as in Figure 3D, is subject to certain amount of shear strain.

**Figure 4:**
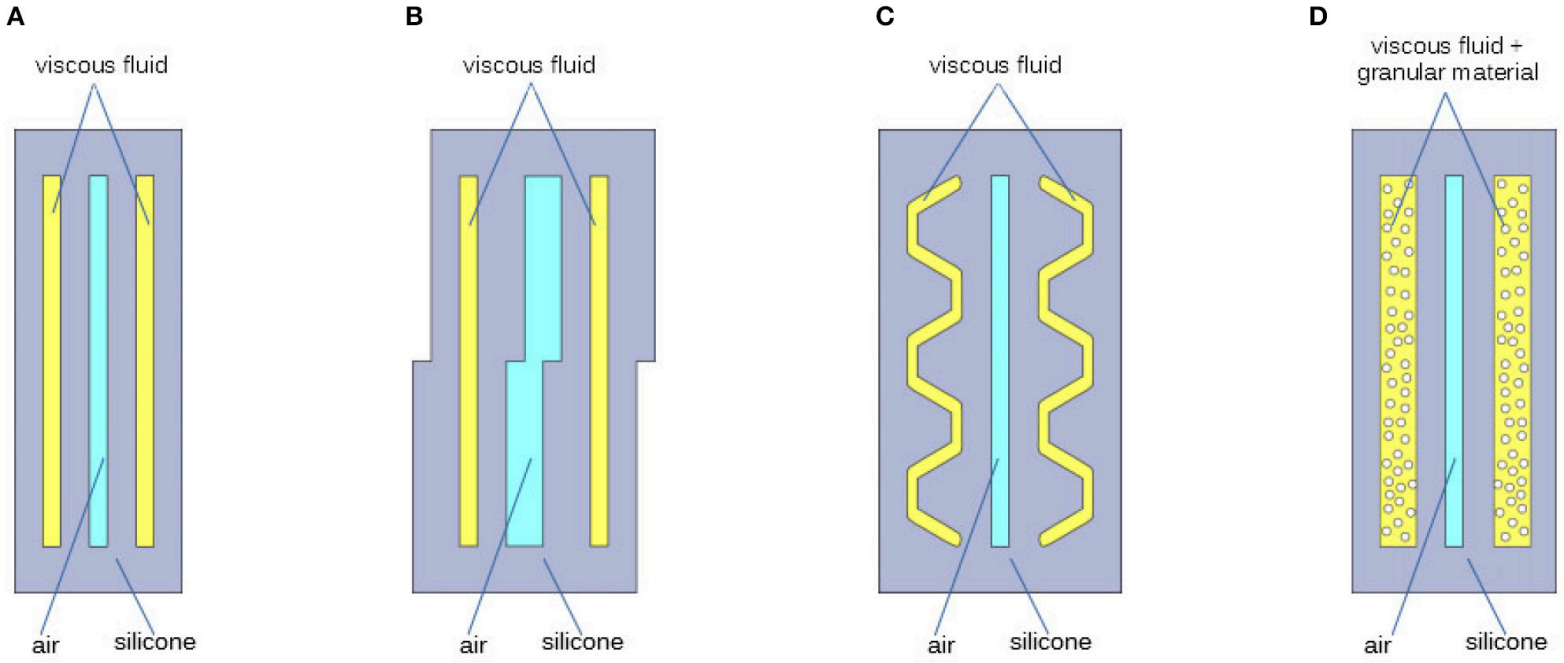
Four cases analyzed for the geometry of the cavity, of the chamber wall, filled with viscous fluid: cavity with simple rectangular shape **(A)**; cavity having walls with uneven stiffness **(B)**; cavity having traits oriented along privileged directions **(C)**; cavity filled with viscous oil and granular material **(D)**.

Were the rectangular chamber be full of viscous fluid, it would be subject to a distribution of dissipative viscous stresses in accordance to

(12) $$\begin{array}{l} \tau =\mu \dot{\gamma }, \end{array}$$

where τ is the viscous shear stress, proportional to the dynamic viscosity μ of the fluid and to the shear strain rate $\dot{\gamma }$. This leads to conceive the simplest possible geometry that could implement a dissipative effect. As shown in Figure 4A, this consists in a *meatus* embedded in the wall of the chamber and filled with viscous oil.

The results of this chamber design are some-how limited by Equations (11), (12), and by the hypotheses under which these were devised. In particular there are three major factors that limit the possible performance, which also suggest three possible ways of improving our design. The first limiting element is that Equation (11) assumes the deformation to be isotropic. A possible way to circumvent this limitation is to act on the stiffness of the walls of the oil chamber, so as to induce strongly asymmetric deformations, and in turn increase the amount of shear strain rate $\dot{\gamma }$. A possible design that implements this feature is that shown in Figure 4B. Looking at Equation (11) in a different way, we can also notice that there are indeed privileged directions along which shear strain rate is maximized. These are the directions corresponding to $\theta =\frac{\pi }{4}+k\frac{\pi }{2}$, i.e., the bisectors of the plane quarters. This suggests that another possible way to increase the shear stress rate is to make the wall of the fluid-storage chamber parallel to these directions. The design displayed in Figure 4C, tries to go in this direction. Finally, the element that most influences the damping action is the coefficient μ of Equation (12). This coefficient depends on the particular type of viscous oil used: considering Newtonian fluids, it is assumed constant. Off-the-shelf silicone oils already span a good range of viscosity^1^, nevertheless, a known method to further increase the equivalent dynamic viscosity of a fluid is to mix in it a suspension of particles (Chong et al., 1971; Macosko and Larson, 1994). From this derives the idea to realize the damping layer by means of granular material immersed in the viscous oil. Its schematic section is represented in Figure 4D.

### 3.1. Simulations

Given the complexity of the problem, in the following we present some simulation analysis of the different cavity designs proposed, to better assess the effective potential of each solution. It is also a way to take into account the main higher order effects that were not considered above, but can be of capital importance in real implementations.

**Figure 5:**
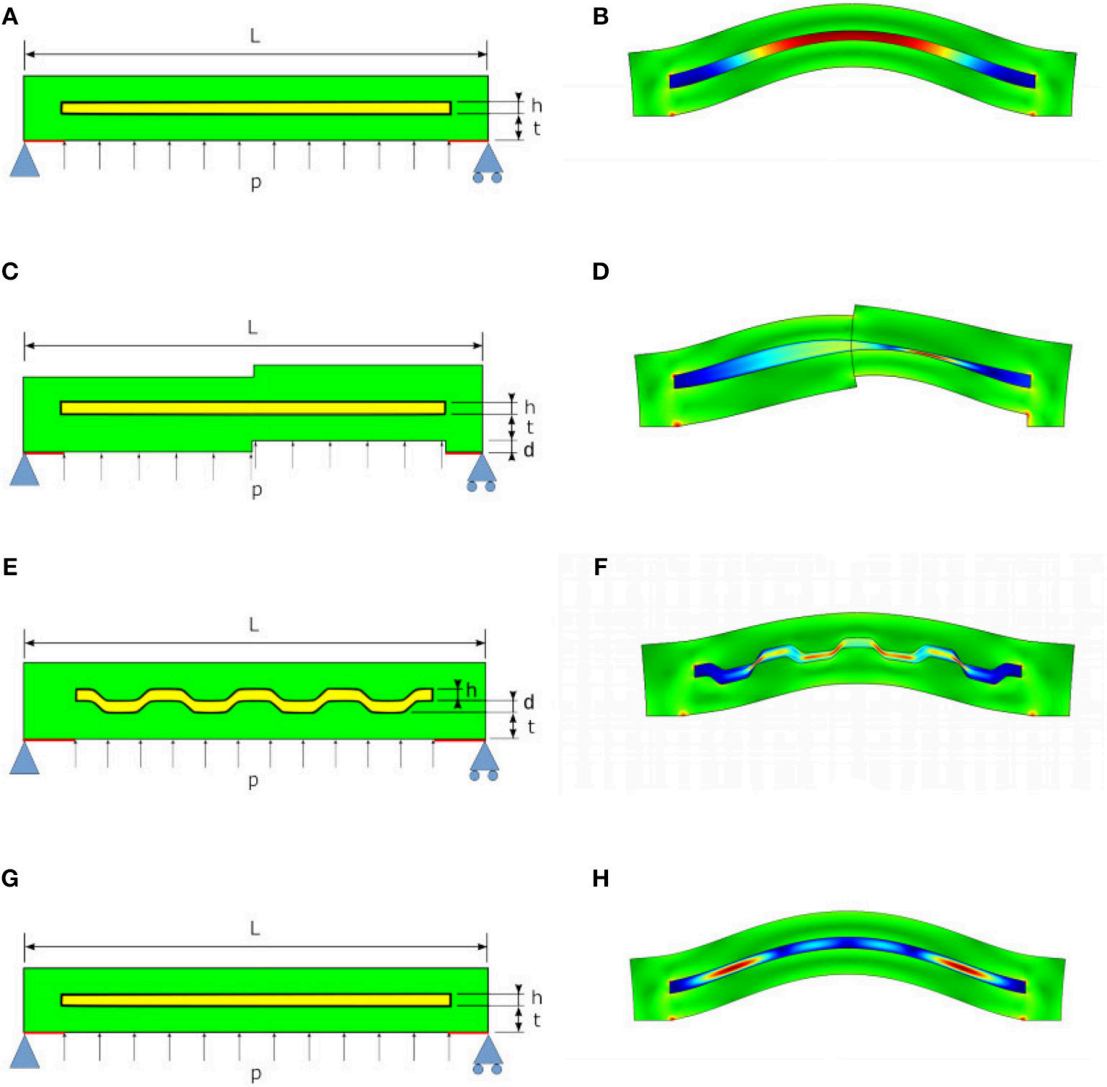
Undeformed (left) and deformed (right) models for the Finite Element Simulation of the proposed chambers depicted in Figure 4. Geometric parameters used for simulation are *L* = 60 mm, *h* = 1.5 mm, *d* = 1.5 mm, and *t* = 3.5 mm. In **A,C,E,G** are also reported the constraints (in light blue) imposed for the modeling of the system and implemented in simulations.

**Figure 6:**
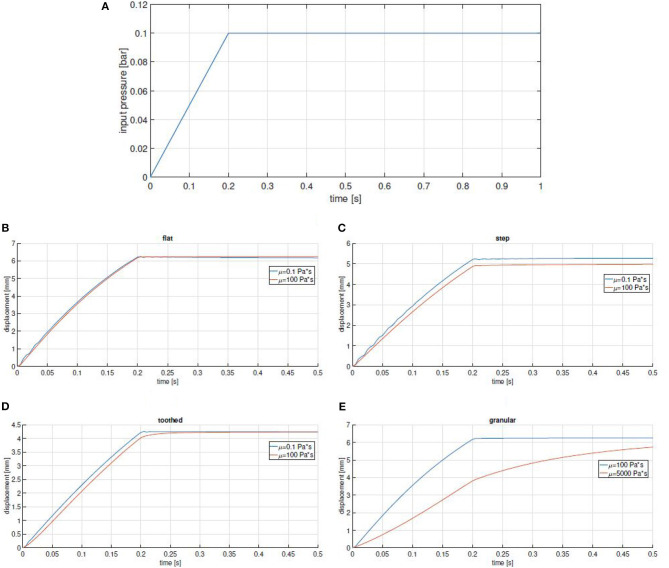
**(A)** Input pressure used in simulation. **(B–E)** Simulation results for all the tested designs reported in Figure 4. Maximum displacement is plotted as a function of time for two extreme values of viscosity. Settling times ratios: 1 **(B,C)**, 0.86 **(D)**, and 0.33 **(E)**.

The effectiveness of our approach to obtain a damping layer has been initially tested through Finite Element Analysis (FEA). Time dependent simulations have been executed in Comsol Multiphysics^2^, using the Fluid-Structure Interaction physics. The built-in silicone material is adopted for the solid part, while a generic oil with custom dynamic viscosity constitutes the liquid component. The models for the FEA of the chambers Figures 4A–C are shown in Figure 5 both in undeformed and deformed configuration. As thickness of the chambers is greater than their width, in first approximation we consider reasonable to simplify the simulation using 2D models. The dimensions of the geometrical parameters defining the chambers are reported in the caption of Figure 5. To reduce the computational effort, only the chamber wall is modeled. It is assumed that the part of the wall that is connected to the rest of the chamber is constrained (red segments in Figure 5). In order to have an isostatic structure, the leftmost node is considered fixed, the others are allowed to move horizontally. The load is induced by the pressurized air in the internal chamber. The simulations are carried out with a time varying pressure *p*, that increase from zero up to 0.1 bar (at 0.2 s), and is kept constant for 0.8 s, as shown in Figure 6A. The simulation of case Figure 4D is particularly tricky, as it should account for the dynamics of a suspension of particles inside the chamber, that interacts in a complex manner with (i) the fluid, (ii) the chamber walls, and (iii) the particles themselves. Following the considerations from Chong et al. (1971), in first approximation, the simulation is performed as in case (a), with a much larger value of viscosity (50 times larger).

The time profile of the maximum displacement of the chambers for two different values of fluid viscosity are reported in Figures 6B–E. It is evident that the flat geometry of chambers (Figure 4A) is totally ineffective, while the behavior of the other ones is affected by changes in oil viscosity. This is likely due to the fact that a more complex geometry favors sliding motions between the two interfaces, generating shear stress in the fluid. However, even in these cases the effect remains still marginal. Case (d), is by far the most promising: as it is possible to see in Figure 6E, the ratio between the settling time of more damped and less damped chambers is between two and three times that of chambers implemented with other approaches.

### 3.2. Experimental Validation

From simulation results, the best solution coincides with the chamber having cavities filled with viscous oil and granular material. Therefore, it has been selected to be tested experimentally^3^.

**Figure 7:**
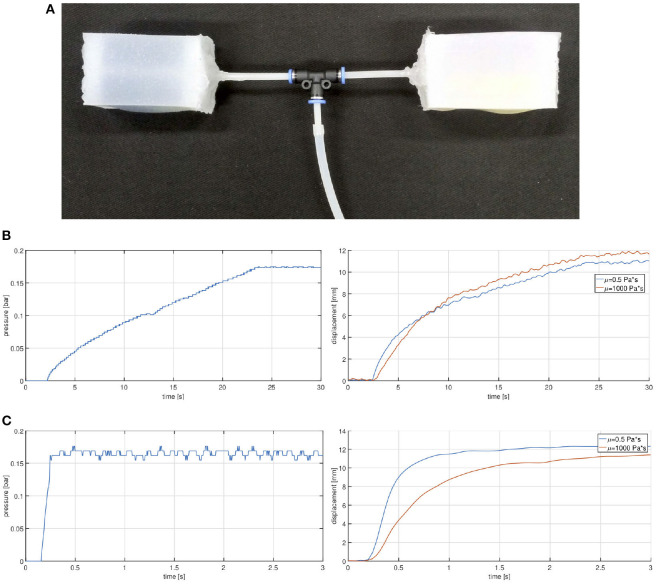
Granular chambers: experimental setup **(A)** and relative results: plot of the supply pressure (left) and of the correspondent measured displacement (right) for the two cases with slow **(B)** and fast **(C)** input.

The experimental setup comprises two identical chambers filled with different viscous silicone oils (μ_*low*_ = 0.5 Pa s and μ_*high*_ = 1, 000 Pa s) and connected in parallel to the same pressure supply (see Figure 7A). Silicone oils are chosen for their chemical compatibility with silicone rubber.

The air supply system relies on an external off-the-shelf air compressor attached to an electro-pneumatic regulator (model SMC ITV2030-31F2BN3-Q). A Custom electronic system with an ADC and DAC converters, is used to interface the pneumatic regulator to a Matlab/Simulink control scheme (more details are available in NMMI^4^ Della Santina et al., 2017). The value of the pressure *p* is intended with respect to the reference external (atmospheric in our case) pressure. Two experiments are executed, corresponding to a fast and a slow input. The pressure is regulated following a reference in the form of a step function (fast input) or a gentle slope (slow input). The experimental setup is video-recorded and analyzed with the Kinovea^5^ software suite. Results are reported in Figure 7: for a slow input (Figure 7B) the two chambers inflate about simultaneously, while for an impulsive pressure (Figure 7C) the high damped chamber exhibits a significant delay with respect to the other.

This result proves the effectiveness of the granular solution as a damping layer to tune the inflation rate of a soft actuator. Unavoidable discrepancies are to be found in the uncertainties due to the employed fabrication techniques. Of course, advancements on this regard would allow to enhance the proposed approach through more accurate and repeatable samples.

### 3.3. On the Effect of Particle Diameter

**Figure 8:**
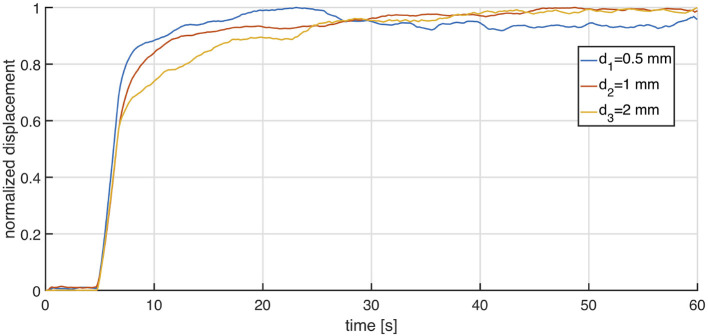
Figure reports experimental results obtained using a box chamber filled with an oil (μ = 1, 000 Pa s) and particles of different size: *d*_1_ = 0.5 mm, *d*_2_ = 1 mm and *d*_3_ = 2 mm. Experiment was conducted adopting a fast inflation, the pressure was regulated to follow a step profile from 0 to 0.1 Pa.

Since the behavior of the liquid-granular suspension is strongly non-linear, it is legitimate to ask what the effect of different particles sizes would be on the system. Although the work of Lewis and Nielsen (1968) suggests that diameter should play a minor role, its results are based on much smaller particles than those used in our study. So we decided to investigate the effect of this particles our selves. For this purpose, three samples of the same box chamber are filled with the same oil (μ = 1, 000 Pa s) but with particles of different size: *d*_1_ = 0.5 mm, *d*_2_ = 1 mm and *d*_3_ = 2 mm. Each of them is tested under fast inflation, where the pressure is regulated to follow a step profile from 0 to 0.1 Pa. Figure 8 reports obtained results in terms of normalized displacement as a function of time. It looks that the effect of particle sizes on damping behavior of the chamber is marginal. This result is in agreement with rheological observations that relative viscosity of suspensions is little affected by particle size, but rather by volumetric fraction (Lewis and Nielsen, 1968). However, for the specific application scope and scale, a certain trend can be inferred, showing that larger particles yield a slightly larger time to reach the steady displacement, thus a more damped dynamics although these differences are only marginal, hereinafter the granular material employed will be of diameter *d* = 2 mm.

## 4. Soft Actuators

**Figure 9:**
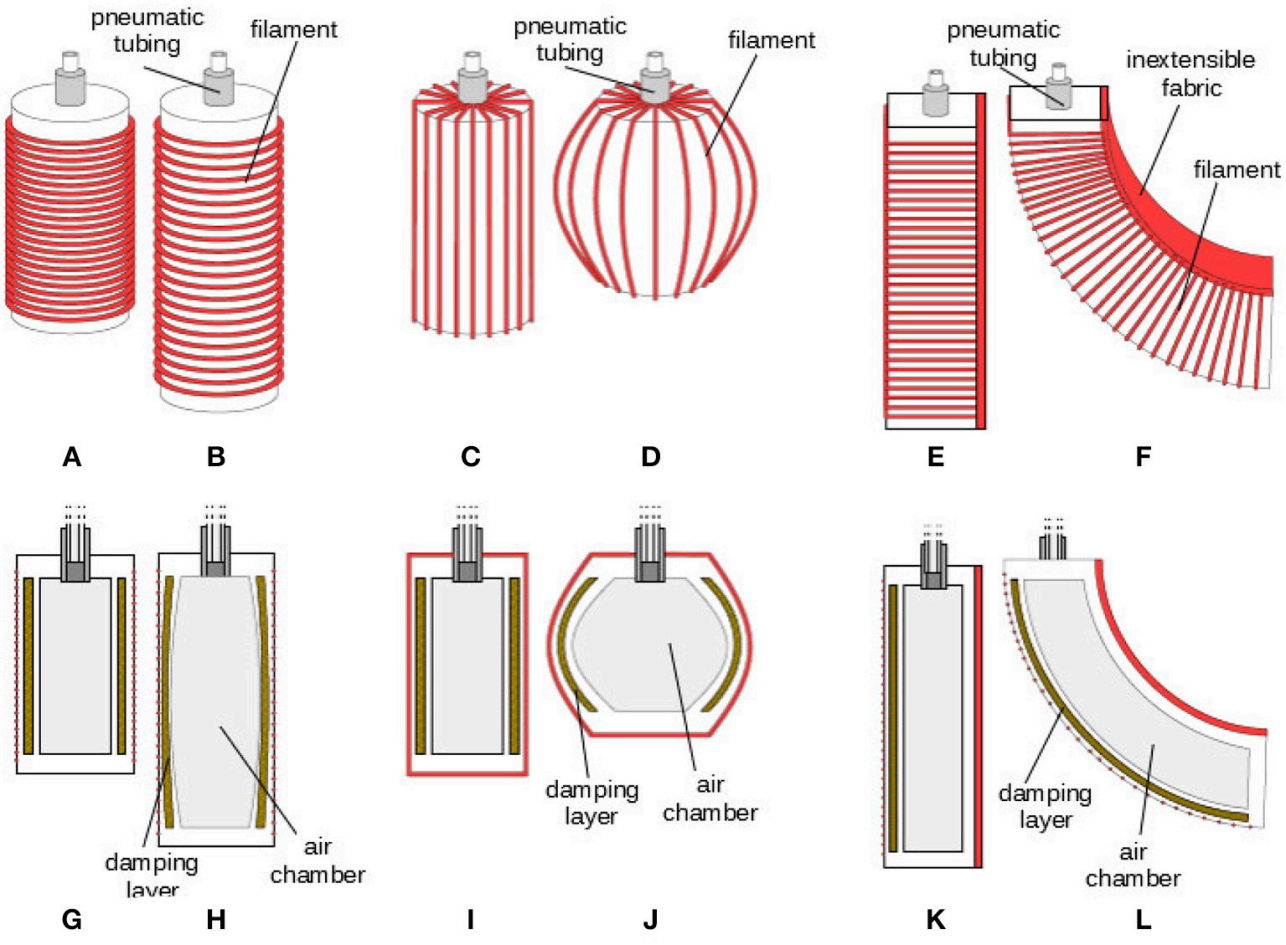
3D concept (top) and section (bottom) of soft damped actuators in their deflated (left) and inflated (right) configuration. Extending **(A,B,G,H)**, contracting **(C,D,I,J)** and bending **(E,F,K,L)** motions are reproduced.

The above principle can be applied to fabricate a variety of soft actuators with different damping factors, as depicted in Figure 9. In this section we explore three different designs to obtain three fundamental movements: extension, Figure 9A, contraction, Figure 9B, and bending, Figure 9C. The mechanism that is used to give to the actuators a main deformation direction is the so-called “fiber reinforcement” (Connolly et al., 2015; Polygerinos et al., 2015b), that exploits the inclusion of inextensible fibers in the actuator design to prevent strain along certain directions. The most common material for wire is polyethylene terephtalate (PET plastic, often called “Polyester”). Different deformation patterns can be obtained for different angles of filament winding. It is worth to be noticed as all the actuators share a similar structure, a similar amount of functional parts and a similar morphology. Referring to Figure 9 all of them present: a *pneumatic tube*, used to pump inside the chamber the pressurized air, a *damping layer* on the chamber walls, an *air chamber* and the *filament* used to imposed the given deformation. Only the bending actuator presents an additional functional component, the *inxestensible fabric material* used to realize the bending movement. In selecting the location of viscous fluid, the main aspect to be considered is the deformation of the cavities. Higher strain implies greater flow of the oil, and so the effect in terms of damping is more pronounced. Therefore, for completeness, a modal analysis would be required to identify these optimal regions, especially if cyclic motions are expected. However, in first approximation a simpler static mechanical analysis may be sufficient, as it is the case of the proposed soft actuators: for cylindrical actuators they are distributed throughout the lateral wall surface, while for bending actuators they are positioned within the side wall that undergoes the biggest elongation. A detailed description of the structure of each actuator, together with details about their fabrication process, is reported in sections 4.2, 4.3, and 4.4 for extending, contracting and bending actuators respectively. General notes about fabrication are reported in section 4.1.

### 4.1. Notes About Fabrication

**Figure 10:**
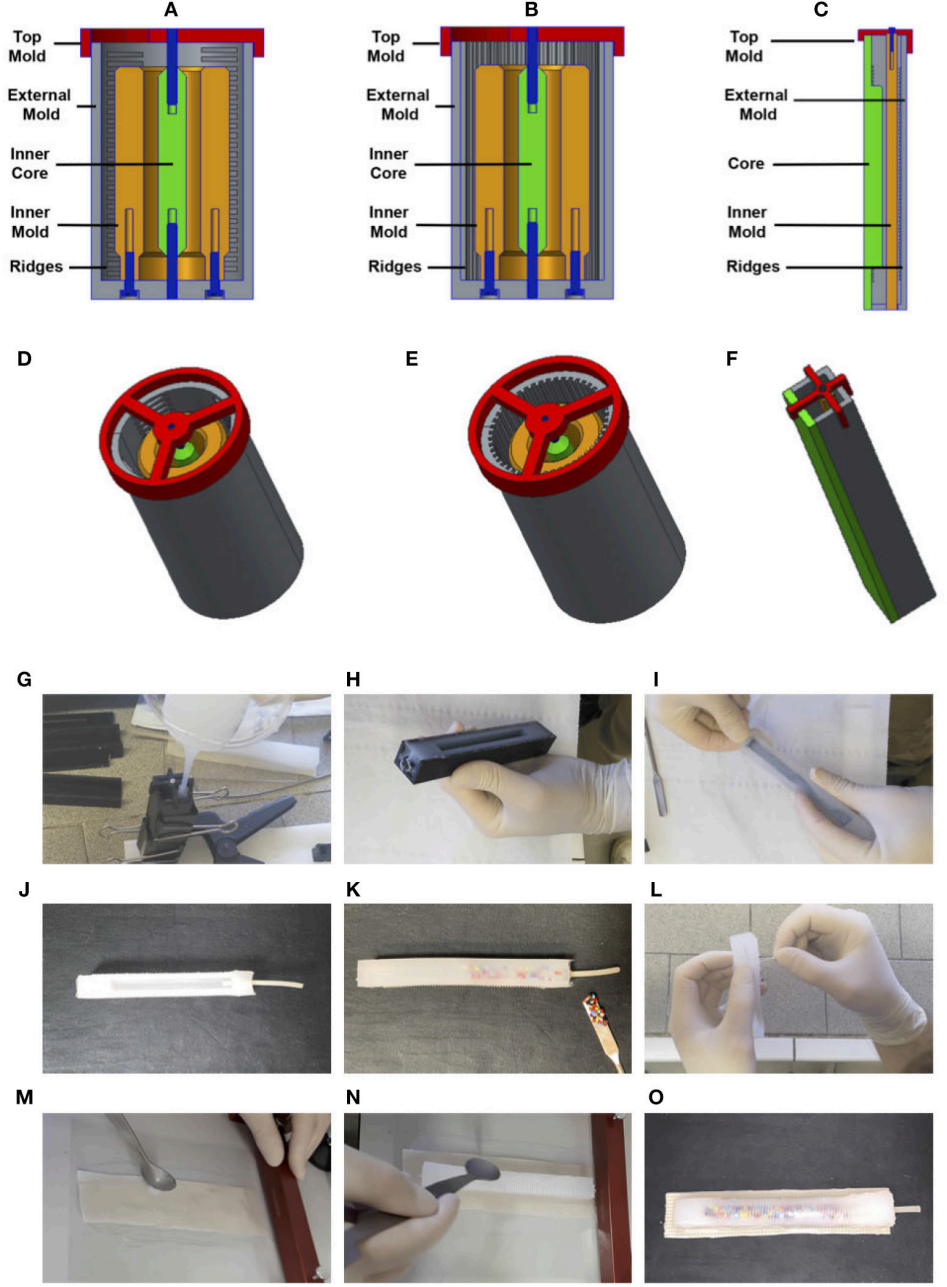
The pictures depict the 2D section **(A–C)** and tridimensional view **(D–F)** of the molds designed to fabricate the three types of soft actuators: extending **(A,D)**, contracting **(B,E)**, bending **(C,F)**. The photo sequence **(G–O)** shows some of the most important phases of the fabrication process of the bending actuator: **(G)** pouring the mold with silicone, **(H)** extracting the silicone frame from the mold, **(I)** extracting the inner core from the silicone frame, **(J)** adding the pneumatic tube in the silicone frame, **(K)** adding of viscous material mixed with the particles, **(L)** winding the wire, **(M)** preparation of the un-extensible material, **(N)** assembly of the inextensible material together with the silicone frame, **(O)** assembled bending actuator.

The fabrication of the soft actuators that are proposed in this work deserves some attention because of their unconventional structure. In Figure 10A schematization of molds used to build actuators is reported. The picture depict the 2D section, Figures 10A–C, and the tridimensional view, Figures 10D–F, of the molds designed to fabricate the three types of soft actuators: extending Figures 10A,D, contracting Figures 10C,E, bending Figures 10C,F.

Multi-material 3D printing would offer a compelling fabrication approach because it allows the fabrication of complex, pre-filled fuidic channels, even for soft elastomers, (MacCurdy et al., 2016). However, this technique is still on its infancy and so far it is limited to a few non-curing liquids designed to be jetted by the printhead, while viscous fluids are not yet available for such integrated process. Hence, the presence of an inner chamber for the pressurized air and an outer hermetic cavity for the viscous oil require a multi-step production process. Additionally, once the silicone gets in contact with the oil, a successful sealing of the cavity requires high care. As depicted in Figure 10 the three molds share a similar structure, indeed all of them present an outer shell, *external mold*, and inner shell, *inner mold*. Molds are closed by a third component, the *top mold*. All the molds present ridges for the winding of reinforcement filament, and have an *inner core* adopted to create internal cameras, that are used to fill inside the compressed air, and for the insertion of the viscous fluid mixed with particles. Details about the different phases of the fabrication process are reported in the photo sequence Figures 10G–O. The photo-sequence is related to the fabrication of the bending actuator, but the same phases apply for both the extending and contracting actuators.

Molds are made of 3D printed parts, that are customized for the three different applications. Material adopted to print the parts is ABS, and the printer used is a Stratasys Fortus 250mc. The actuators are built by molding EcoFlex 00-30 silicone (SmoothOn^6^, elastic modulus *E* = 125 kPa).

### 4.2. Extending Actuators

A logical scheme of the extending actuator is represented in Figures 9A,B,G,H. The chamber has a cylindrical shape. The fibers for the reinforcement are winded around the chamber in a circumferential direction in order to obtain an extension movement when the chamber is inflated. The cylindrical actuators (shown in Figure 10B) are realized through an intermediate mold (orange) and an external mold (gray) and a core (green). The external mold is divided into two halves that are screwed to the intermediate mold. The internal core is located via two pins (blue) one connected to a top (red) and the other connected to the external frame. The employment of fiber reinforcement is adopted to obtain specific deformation patterns (axial or radial). For this end some ridges are designed on the external mold in order to generate on the silicone body the notches where the thread is winded.

### 4.3. Contracting Actuators

A logical scheme of the contracting actuator is represented in Figures 9C,D,I,J. The fibers for the reinforcement are winded around the chamber in a longitudinal direction in order to obtain a contraction movement. The fabrication mold and process is similar to the one described for Extending Actuators. In this case the employment of fiber reinforcement is adopted to obtain a radial deformation pattern. For this end some ridges are designed on the external mold in order to generate on the silicone body the notches where the thread is winded.

### 4.4. Bending Actuators

A logical scheme of the bending actuator is represented in Figures 9E,F,K,L. The fibers for the reinforcement are winded around the chamber in a circumferential direction in order to avoid its enlargement. One of the faces of the actuator embeds a un-extensible, yet flexible element. This concept results in a large bending deformation. The finger mold (Figure 10C) is inspired by the PneuFlex actuator^7^ composing the RBO Hand (Deimel and Brock, 2013). It includes a single air channel along its entire length and undergoes a continuum bending when inflated thanks to the presence of an inextensible fabric on one face. The main difference that allows to use the principle proposed in this paper is a cavity to hold the viscous material on the back, which is the most elongating part.

## 5. Experimental Validation

**Figure 11:**
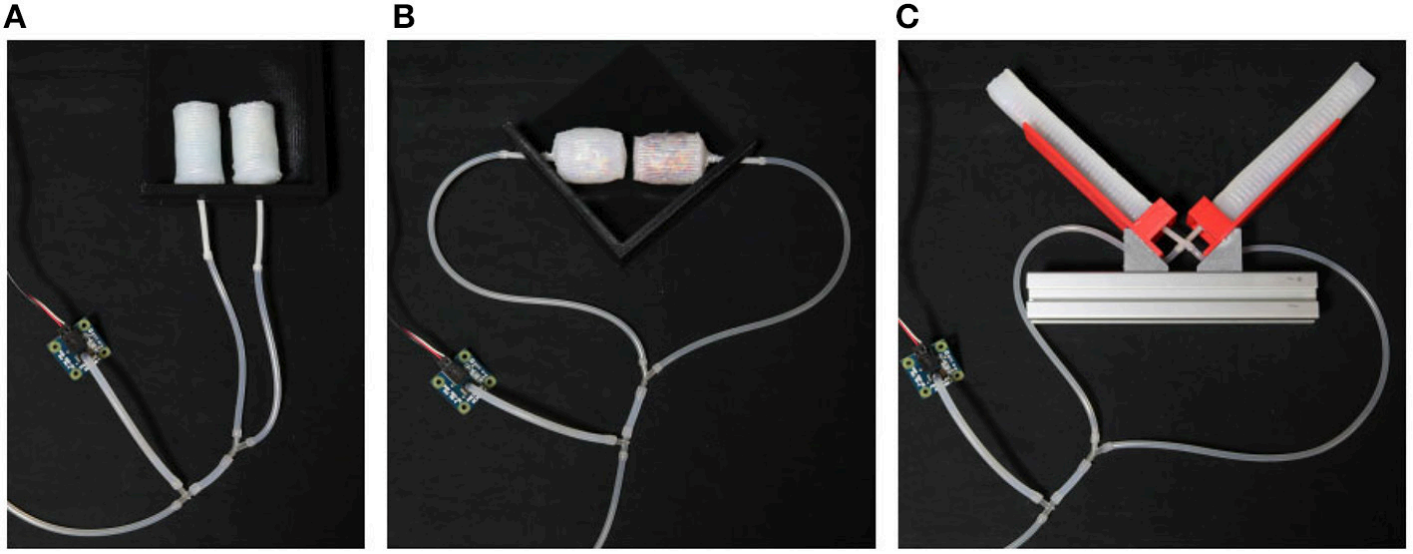
Experimental setup for the characterization of extending **(A)**, contracting **(B)**, and bending **(C)** actuators.

The same values as above are used for the fluids viscosity: μ_*high*_ = 1, 000 Pa s for high-damped sample and μ_*low*_ = 0.5 Pa s for low-damped sample. It is worth noting that the quantities of oil/granular materials are balanced in weight among equivalent actuators, because different quantities of the infill material could affect both the stiffness and damping responses. The resulting weight of each sample is 50, 50, and 55 g for extending, contracting and bending actuators, respectively. For each kind of soft actuator, performances of the two differently damped samples are compared in case of slow (inflation duration is about 30 s) and fast (inflation duration is about 0.5 s) pressurization. During experiments two samples with same geometry but different damping layers are connected in parallel to the same pressure source. A syringe pump is used to supply the air and the pressure is measured through the Phidgets pressure sensor 1115^8^. Experimental setups are shown in Figure 11 for all the three kinds of soft actuators.

### 5.1. Extending Actuators

**Figure 12:**
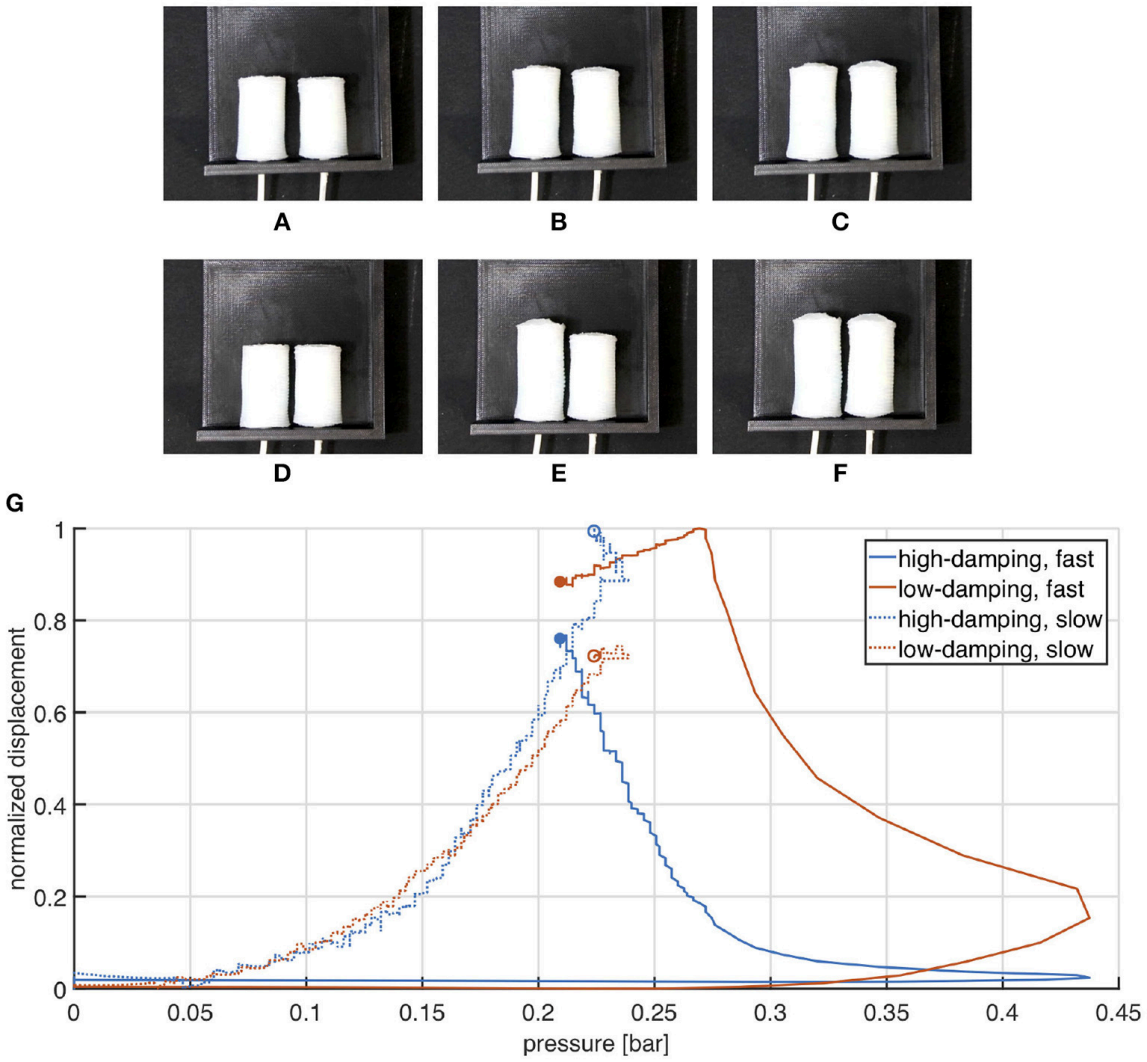
Comparison between high and low damping extending actuators undergoing slow **(A–C)** and fast **(D–F)** pressurization. Frames **(A–C)** are captured at times 0, 30, and 60 s, while **(D–F)** refer to times 0, 0.5, and 5 s. Normalized displacement is plotted as a function of the measured pressure. **(G)** Experimental validation of the extending actuator. Marked points represent the final state.

Results of the experimental characterization are represented in Figure 12. The two extending samples are arranged next to each other along two parallel directions. The ends connected to the pneumatic line are fixed to a support, while the others are left free to move. It is evident that both soft actuators extend almost simultaneously under slow actuation (Figures 12A–C), while in case of fast pressurization the high-damped sample lags behind the other. By the way, in the end the two samples reach almost the same elongation, meaning that their stiffness is maintained comparable. These arguments are reflected in the chart reported in Figure 12G, where the two actuators exhibit a similar response under slow actuation (which means similar stiffness), while their behaviors significantly diverge for a high inflation rate (as a consequence of substantially different damping). It is worth noting that in the latter case a spike is measured in the pressure signal at the end of the inflation phase. It is supposedly due to the inertial contribution produced by the syringe actuation and it causes the pressure drop visible in the correspondent plot.

### 5.2. Contracting Actuators

**Figure 13:**
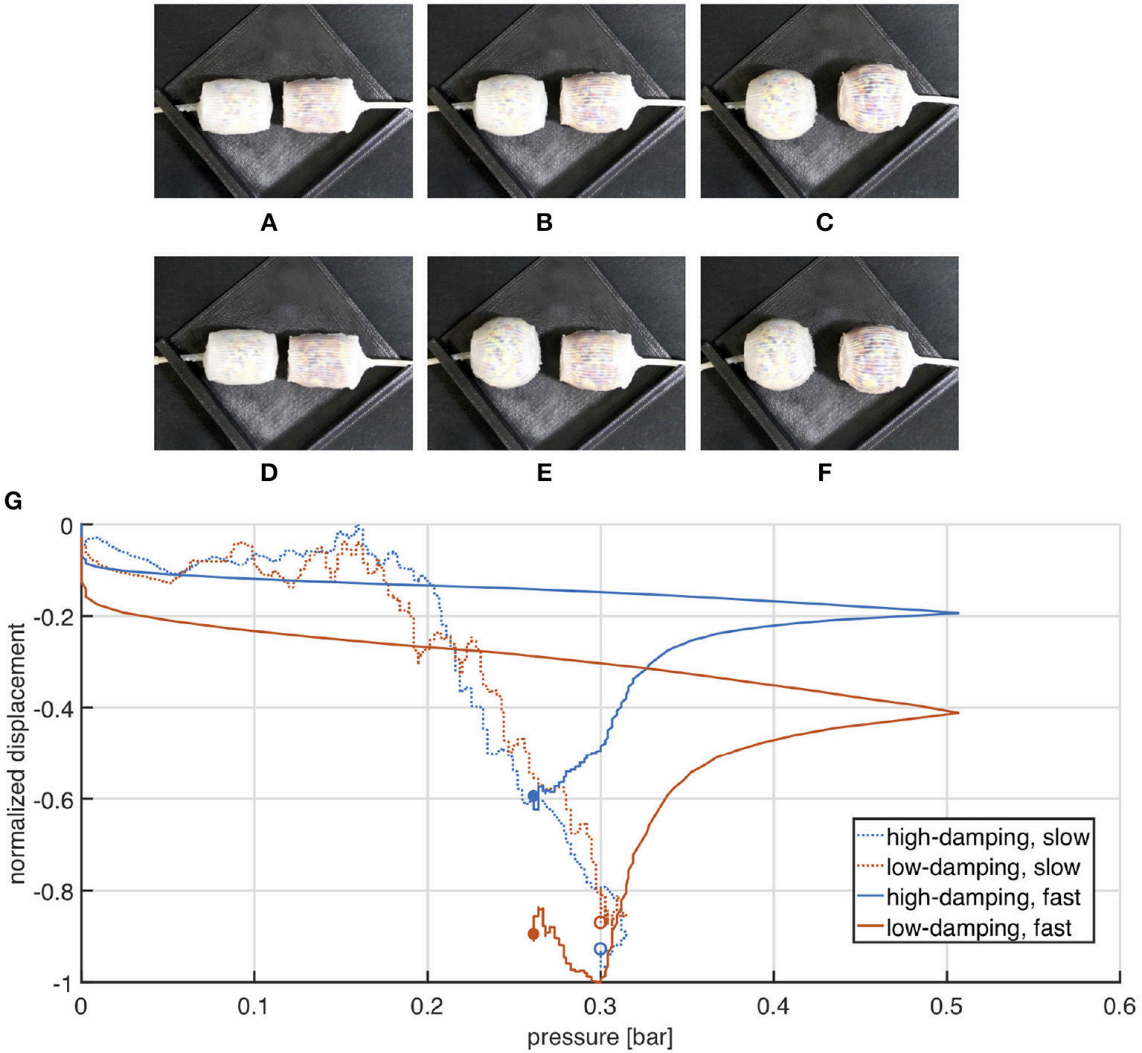
Comparison between high and low damping contracting actuators undergoing slow **(A–C)** and fast **(D–F)** pressurization. Frames **(A–C)** are captured at times 0, 30, and 60 s, while **(D–F)** refer to times 0, 0.5, and 5 s. Normalized displacement is plotted as a function of the measured pressure. **(G)** Experimental validation of the contracting actuator. Marked points represent the final state.

Results of the experimental characterization are represented in Figure 13. The soft actuators are counter-posed along the same line, with their pneumatic tubing pointing outwards.

When the chambers are slowly pressurized they contract simultaneously and maintain a nearly symmetric configuration (Figures 13A–C). Conversely, a significant asymmetry is produced by a fast actuation: the low damped actuator moves significantly faster than the other (Figures 3D–F). The plot in Figure 13G shows that the pressure strike is still considerable under fast actuation, nevertheless the behavior of the two actuators is as expected: their curves almost overlap for slow inflation, while they move noticeably away each from the other at fast pressurization.

### 5.3. Bending Actuators

**Figure 14:**
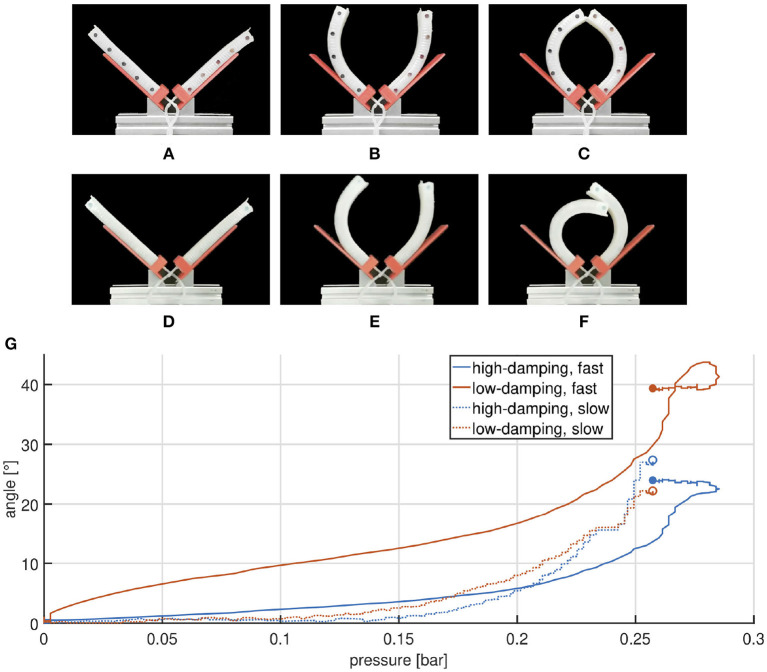
Comparison between high and low damping bending actuators undergoing slow **(A–C)** and fast **(D–F)** pressurization. Frames **(A–C)** are captured at times 0, 30, and 60 s, while **(D–F)** refer to times 0, 0.5, and 5 s. Angular displacement is plotted as a function of the measured pressure **(G)**. Experimental validation of the bending actuator. Marked points represent the final state.

For the experimental characterization the two samples are placed on a proper designed support, one in front of the other, as shown in Figure 11C. Also in this case the reciprocal behavior of the two chambers shows a clear difference between slow and fast actuation. More in detail, a slow pressurization produces a simultaneous closure of the bending chambers (Figures 14A–C), instead when a fast input is introduced, the high-damped chamber takes more time than the other to close and stops on top of it (Figures 14D–F). These considerations are confirmed from the plot in Figure 14G.

## 6. Possible Application Examples

Presented soft actuators with different damping properties can be combined to realize under-actuated systems able to switch their behavior according to the actuation rate. Of course, some theoretical limitatios to the applicability of the method derive from employed viscoelastic materials and pneumatic actuation. A viscoelastic behavior implies a combination of an ideal elastic response and an ideal viscous response. Considering a sinusoidal deformation, as it occurs in a DMA, for an ideal elastic material, the stress and strain are in phase, and the phase shift is null, while for an ideal viscous material, the stress and strain are 90° out of phase. Therefore, given a baseline design of a soft chamber, ideally a maximum phase lag of 90° could be achieved. Actually, differences of phase achievable with the proposed method are much smaller due to practical limitations. These are mainly related to the fabrication process of the chambers, which relies on manual operations, inherently inaccurate and subject to uncertainties. For instance, an evaluation obtained from the identification of the high damped box actuator with a second order system provides a phase shift of about 45° accounting for an attenuation of −3 dB at frequency of about 0.5 Hz. Phase lags outside the available range need to be addressed with a different approach. An example is reported in Di Lallo et al. (2018), where a hinged mechanism is exploited to produce a 180° phase shift between two modules of a soft articulated robot. Moreover, as viscous component increases, hysteresis is exacerbated, making energy dissipation a possible issue for applications where efficiency or thermal management constitute critical aspects. Other restrictions about the proposed method concern robots undergoing periodic motions, where the frequency bandwidth is limited by several factors. On one side, input frequency is subject to an upper bound due to the magnitude attenuation of the response of the system (modeled as a second order system) beyond its natural frequency and to the maximum flow rate allowed by pneumatic actuation. On the other side, it should be kept away from the natural frequencies of the system, in order to avoid any resonance phenomenon. On this regard, modal and frequency analyses would be beneficial to have a raw expectation about the system behavior and to choose optimal excitation frequency ranges. For this purpose, more accurate models are needed, that would help to predict the time-varying material profiles of soft designs. Furthermore, it would be interesting to investigate in future work the effects of some key parameters related to pneumatic actuation, such as the chamber size or the length of the feeding line. For micro-robots, that imply movement of small volumes of air, larger pressure ramp rates could be needed. Similarly, the effectiveness of the method is expected to decrease as involved volume of air increases, because of the compressibility of the air. Consequently, larger systems could require taking into account alternative incompressible fluids (e.g., water) for actuation. In the following, some proof-of-concept application examples are shown.

### 6.1. Two-Fingered Soft Gripper

**Figure 15:**
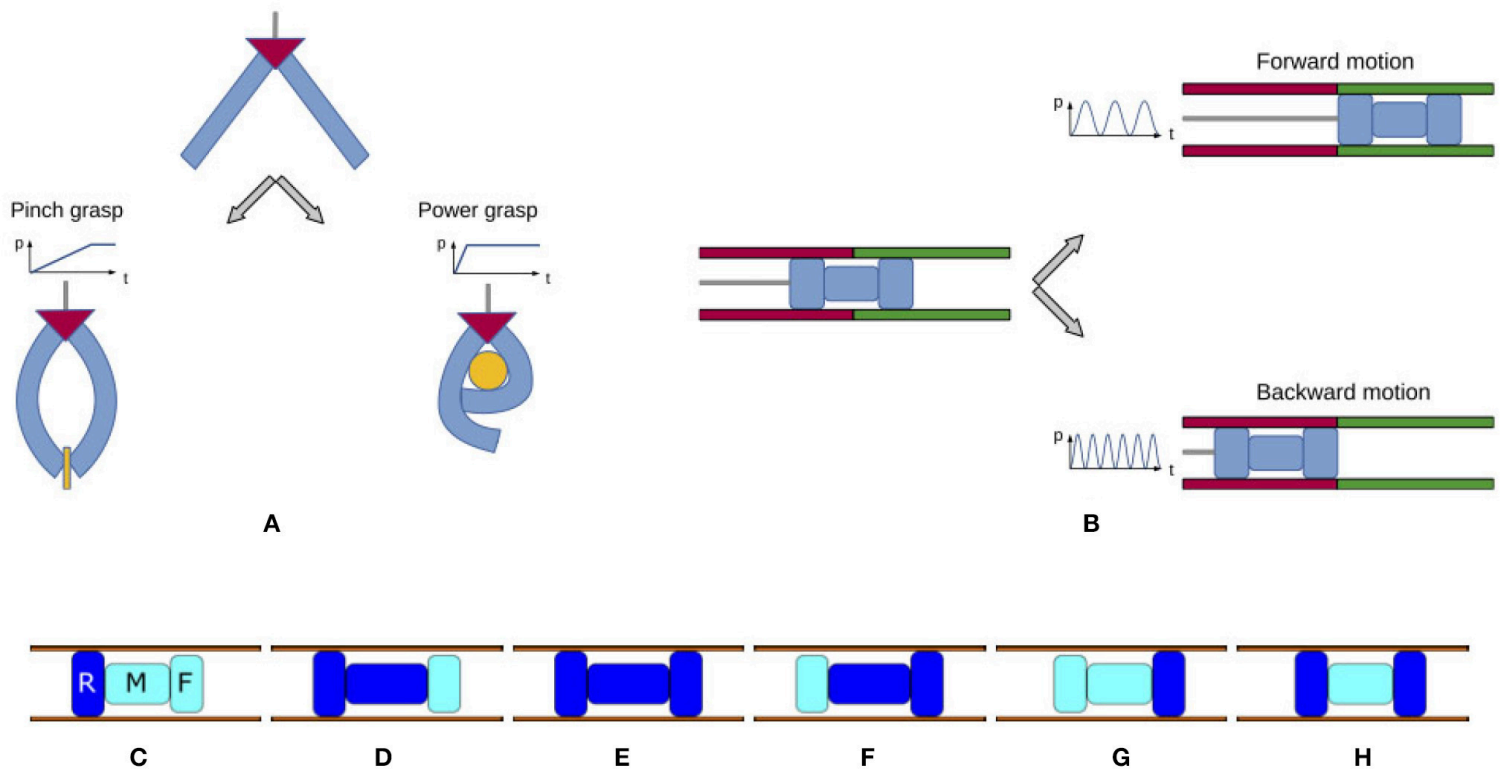
Proof-of-concept applications: soft gripper able to perform pinch and power grasp, inspired by Piazza et al. (2016) **(A)**; soft worm-like robot able to move forward and backward, inspired by Di Lallo et al. (2018) **(B)**. **(C–H)** Evolution of the three modules composing an inchworm during a forward gait cycle. Inflated chambers are dark blue, deflated chambers are light cyan.

Taking inspiration from the work in Piazza et al. (2016), it is possible to use two bending actuators to realize an underactuated two-fingered soft gripper, able to perform precision and power grasps. In the envisioned solution depicted in Figure 15A the two fingers are connected in parallel to the same pressure source and different closure patterns can be obtained by modulating the inflation rate. As discussed above, a slow pressurization elicits a symmetric closure of the fingers. It corresponds to an advantageous configuration for pinch grasp, useful to precisely grasp small and light objects. On the other side, when a fast input is provided, a finger closes on top of the other, favoring an enveloping grasp (power grasp), better suited for large, heavy objects. Therefore, the two grasp behaviors are complementary in widening the gripper flexibility.

### 6.2. Inchworm Soft Robot

Another classical application of pneumatic systems is that of locomotion of worm-like robots, where the absence of a solid structure often limits the range of available actuators. As a particular case, inchworm robots are appealing for pipe maintenance and diagnostics due to their shape and operational mechanism (Bertetto and Ruggiu, 2001; Sabzehmeidani et al., 2011; Gargade and Ohol, 2016). In fact, as discussed in (Di Lallo et al., 2018), inchworm robots substantially require two kinds of forces to implement propulsion: an impelling force and a holding force. The former is the force to push the robot forward, while the latter serves to fix the robot against the pipe wall. The right sequencing of these forces produces propulsion in a pipe. To generate such forces, an inchworm system can be modeled by a set of at least three modules: rear (R) and front (F) modules aim at holding the robot against the pipe walls, while the middle (M) one is responsible for the impelling movement. Their activation in the right order produces a forward gait cycle, as shown in Figures 15C–H. Playing the same cycle in reverse, on the other hand, will yield backward locomotion. In our previous work (Di Lallo et al., 2018) we showed that both these behaviors can be achieved using a single feeding line, common to the three modules. In particular, a proper design of the mechanical properties of the three chambers allows to switch between forward or backward locomotion by simply modulating the frequency of the pressure input. Thanks to the contribution of present work, the same concepts could be transferred in the field of soft continuum robots. The combination of the presented soft actuators with opportunely designed stiffness and damping features would enable to assembly an under-actuated soft continuum inchworm robot, able to switch the crawling direction by tuning the frequency of the input source, as depicted in Figure 15B.

## 7. Conclusions

The rationale behind this study derives from an under-actuated control strategy for soft robots and fits in the perspective of a dynamic morphological computation. In fact, this paper proposes an experimentally validated solution to the problem of designing soft inflatable chambers with different damping properties by adding suitable cavities with viscous fluid and granular media. The principle has been applied to realize differently damped soft continuum actuators that can elongate, contract, and bend. The proposed actuators have been experimentally validated, and an overview about the fabrication process is provided along with the description of their structure and working principle. Finally, some proof-of-concept applications are suggested, where the combination of multiple such actuators allows to produce an under-actuated system that is able to switch among different dynamical behaviors by simply acting on the actuation rate. Future work will investigate the application of the proposed technology to mobile robots and soft continuum end-effectors.

## Author Contributions

AD executed research, performed experiments, and simulations. MC, GG, and MGar supervised research. All the above mentioned authors analyzed the data and wrote the manuscript. AB and MGab conceived research and contributed expertise.

### Conflict of Interest Statement

The authors declare that the research was conducted in the absence of any commercial or financial relationships that could be construed as a potential conflict of interest.

## Footnotes

^1^Commercially available silicone oils reach viscosity values of 1000 Pa s.

^2^COMSOL Multiphysics Modeling Software, https://www.comsol.com/

^3^Concerning the fabrication of the box actuators, the whole mold consists of an outer frame composed of four separate layers and three internal cores. After unmolding, the result of the process is a chamber with three openings: the external ones are used to pour the damping oil and then are closed, while the air tube is attached to the center chamber. A silicone adhesive is employed as sealing agent. For more details on the fabrication please refer to section 4.

^4^Natural Machine Motion Initiative, https://www.naturalmachinemotioninitiative.com/

^5^Kinovea, http://www.kinovea.org

^6^Smooth-On, Inc. -Mold Making & Casting Materials-Rubbers, Plastics, Foams & More!, https://www.smooth-on.com/

^7^Robotics: PneuFlex actuator, https://www.robotics.tu-berlin.de

^8^Phidgets Inc. - Products for USB Sensing and Control, https://www.phidgets.com/

## References

[B1] Bertetto A. M. Ruggiu M. (2001). In-pipe inch-worm pneumatic flexible robot, in Advanced Intelligent Mechatronics, 2001. Proceedings. 2001 IEEE/ASME International Conference on Vol. 2 (IEEE), 1226–1231.

[B2] Booth J. Case J. White E. Shah D. Kramer-Bottiglio R. (2018). An addressable pneumatic regulator for distributed control of soft robots, in IEEE International Conference on Soft Robotics (RoboSoft) (Livorno).

[B3] Boyraz P. Runge G. Raatz A. (2018). An overview of novel actuators for soft robotics, in Actuators, Vol. 7 (Multidisciplinary Digital Publishing Institute), 48.

[B4] Calisti M. Giorelli M. Levy G. Mazzolai B. Hochner B. Laschi C.. (2011). An octopus-bioinspired solution to movement and manipulation for soft robots. Bioinspirat. Biomimet. 6:036002. 10.1088/1748-3182/6/3/036002 21670493

[B5] Chong J. Christiansen E. Baer A. (1971). Rheology of concentrated suspensions. J. Appl. Polymer Sci. 15, 2007–2021. 10.1002/app.1971.070150818

[B6] Chou C.-P. Hannaford B. (1996). Measurement and modeling of mckibben pneumatic artificial muscles. IEEE Trans. Rob. Autom. 12, 90–102. 10.1109/70.481753

[B7] Connolly F. Polygerinos P. Walsh C. J. Bertoldi K. (2015). Mechanical programming of soft actuators by varying fiber angle. Soft Rob. 2, 26–32. 10.1089/soro.2015.0001

[B8] Cowan L. S. Walker I. D. (2008). “Soft” continuum robots-the interaction of continuous and discrete elements, in ALIFE (Winchester), 126–133.

[B9] Deimel R. Brock O. (2013). A compliant hand based on a novel pneumatic actuator, in Robotics and Automation (ICRA), 2013 IEEE International Conference on (IEEE), 2047–2053.

[B10] Della Santina C. Piazza C. Gasparri G. M. Bonilla M. Catalano M. G. Grioli G. (2017). The quest for natural machine motion: an open platform to fast-prototyping articulated soft robots. IEEE Rob. Autom. Mag. 24, 48–56. 10.1109/MRA.2016.2636366

[B11] Di Lallo A. Catalano M. Garabini M. Grioli G. Gabiccini M. Bicchi A. (2018). A novel approach to under-actuated control of fluidic systems, in Robotics and Automation (ICRA), 2018 IEEE International Conference on (Brisbane: IEEE).

[B12] Ertas B. H. Mook J. T. Bellardi J. J. (2017). Fluid-Filled Damper for Gas Bearing Assembly. U.S. Patent App. 15/131,097.

[B13] Fincan M. (2015). Assessing Viscoelastic Properties of polydimethylsiloxane (pdms) Using Loading and Unloading of the Macroscopic Compression Test. Thesis, MS in Materials Science and Engineering.

[B14] Galantini F. Carpi F. Gallone G. (2013). Effects of plasticization of a soft silicone for dielectric elastomer actuation. Smart Mater. Struct. 22, 104020 10.1088/0964-1726/22/10/104020

[B15] Gargade A. Ohol S. (2016). Development of in-pipe inspection robot. IOSR J. Mechan. Civil Eng. 13, 64–72. 10.9790/1684-1304076472

[B16] Greer J. D. Morimoto T. K. Okamura A. M. Hawkes E. W. (2018). A soft, steerable continuum robot that grows via tip extension. Soft Robot. 6, 95–108. 10.1089/soro.2018.0034 30339050

[B17] Jones P. J. (2000). Fluid damper including flexible damping plate. U.S. Patent 6,045,328.

[B18] Katzschmann R. K. DelPreto J. MacCurdy R. Rus D. (2018). Exploration of underwater life with an acoustically controlled soft robotic fish. Sci. Rob. 3:eaar3449 10.1126/scirobotics.aar3449 33141748

[B19] Kim S. Laschi C. Trimmer B. (2013). Soft robotics: a bioinspired evolution in robotics. Trends Biotechnol. 31, 287–294. 10.1016/j.tibtech.2013.03.002 23582470

[B20] Laschi C. Cianchetti M. Mazzolai B. Margheri L. Follador M. Dario P. (2012). Soft robot arm inspired by the octopus. Adv. Robot. 26, 709–727. 10.1163/156855312X626343

[B21] Lee J. Go E. Choi W. Kim W. Cho K. (2016). Development of soft continuum manipulator with pneumatic and tendon driven actuations, in 2016 13th International Conference on Ubiquitous Robots and Ambient Intelligence (URAI) (Xi'an), 377–379.

[B22] Lewis T. Nielsen L. (1968). Viscosity of dispersed and aggregated suspensions of spheres. Trans. Soc. Rheol. 12, 421–443. 10.1122/1.549114

[B23] Liu J. (2009). Analysis of a porous elastic sheet damper with a magnetic fluid. J. Tribol. 131:021801 10.1115/1.3075870

[B24] MacCurdy R. Katzschmann R. Kim Y. Rus D. (2016). Printable hydraulics: a method for fabricating robots by 3d co-printing solids and liquids, in 2016 IEEE International Conference on Robotics and Automation (ICRA) (Stockholm).

[B25] Macosko C. W. Larson R. G. (1994). Rheology: Principles, Measurements, and Applications. Wiley.

[B26] McGuire D. P. (1996). Hybrid Fluid and Elastomer Damper. U.S. Patent 5,501,434.

[B27] McGuire D. P. (2000). Fluid and Elastomer Damper. U.S. Patent 6,092,795.

[B28] Menard K. P. (2008). Dynamic Mechanical Analysis: A Practical Introduction. CRC Press.

[B29] Mosadegh B. Polygerinos P. Keplinger C. Wennstedt S. Shepherd R. F. Gupta U. (2014). Pneumatic networks for soft robotics that actuate rapidly. Adv. Funct. Mater. 24, 2163–2170. 10.1002/adfm.201303288

[B30] Napp N. Araki B. Tolley M. T. Nagpal R. Wood R. J. (2014). Simple passive valves for addressable pneumatic actuation, in Robotics and Automation (ICRA), 2014 IEEE International Conference on (Hong Kong: IEEE), 1440–1445.

[B31] Onal C. D. Chen X. Whitesides G. M. Rus D. (2017). Soft mobile robots with on-board chemical pressure generation, in Robotics Research eds Christensen H. I. Khatib O. (Flagstaff: Springer), 525–540.

[B32] Pfeifer R. Iida F. Gómez G. (2006). Morphological computation for adaptive behavior and cognition, in International Congress Series, Vol. 1291, (Kitakyushu: Elsevier), 22–29.

[B33] Piazza C. Della Santina C. Catalano M. Grioli G. Garabini M. Bicchi A. (2016). Softhand pro-d: Matching dynamic content of natural user commands with hand embodiment for enhanced prosthesis control, in Robotics and Automation (ICRA), 2016 IEEE International Conference on (IEEE), 3516–3523.

[B34] Polygerinos P. Wang Z. Galloway K. C. Wood R. J. Walsh C. J. (2015a). Soft robotic glove for combined assistance and at-home rehabilitation. Rob. Auton. Syst. 73, 135–143. 10.1016/j.robot.2014.08.014

[B35] Polygerinos P. Wang Z. Overvelde J. T. Galloway K. C. Wood R. J. Bertoldi K. (2015b). Modeling of soft fiber-reinforced bending actuators. IEEE Trans. Rob. 31, 778–789. 10.1109/TRO.2015.2428504

[B36] Roche E. T. Horvath M. A. Wamala I. Alazmani A. Song S.-E. Whyte W.. (2017). Soft robotic sleeve supports heart function. Sci. Trans. Med. 9:eaaf3925. 10.1126/scitranslmed.aaf3925 28100834

[B37] Rossiter J. Walters P. Stoimenov B. (2009). Printing 3d dielectric elastomer actuators for soft robotics, in Electroactive Polymer Actuators and Devices (EAPAD) 2009, Vol. 7287, (San Diego: International Society for Optics and Photonics), 72870H.

[B38] Rus D. Tolley M. T. (2015). Design, fabrication and control of soft robots. Nature 521:467. 10.1038/nature14543 26017446

[B39] Sabzehmeidani Y. Mailah M. Hussein M. (2011). Modelling and control of a piezo actuated micro robot with active force control capability for in-pipe application. Int. J. Model. Identif. Control 13, 301–309. 10.1504/IJMIC.2011.041785

[B40] Schmidt W. E. McGuire D. P. (1994). Fluid-and-Elastomer Support Device. U.S. Patent 5,374,039.

[B41] Shepherd R. F. Ilievski F. Choi W. Morin S. A. Stokes A. A. Mazzeo A. D.. (2011). Multigait soft robot. Proc. Natl. Acad. Sci. U.S.A. 108, 20400–20403. 10.1073/pnas.1116564108 22123978 PMC3251082

[B42] Takeichi M. Suzumori K. Endo G. Nabae H. (2017). Development of giacometti arm with balloon body. IEEE Rob. Autom. Lett. 2, 951–957. 10.1109/LRA.2017.2655111

[B43] Tan K. K. Lee T. H. Huang S. (2007). Precision Motion Control: Design and Implementation. Springer Science & Business Media.

[B44] Tolley M. T. Shepherd R. F. Mosadegh B. Galloway K. C. Wehner M. Karpelson M. (2014). A resilient, untethered soft robot. Soft Rob. 1, 213–223. 10.1089/soro.2014.0008

[B45] Vanderborght B. Albu-Schäffer A. Bicchi A. Burdet E. Caldwell D. G. Carloni R. (2013). Variable impedance actuators: a review. Rob. Auton. Syst. 61, 1601–1614. 10.1016/j.robot.2013.06.009

[B46] Wehner M. Tolley M. T. Mengüç Y. Park Y.-L. Mozeika A. Ding Y. (2014). Pneumatic energy sources for autonomous and wearable soft robotics. Soft Rob. 1, 263–274. 10.1089/soro.2014.0018

[B47] Wehner M. Truby R. L. Fitzgerald D. J. Mosadegh B. Whitesides G. M. Lewis J. A.. (2016). An integrated design and fabrication strategy for entirely soft, autonomous robots. Nature 536:451. 10.1038/nature19100 27558065

[B48] White F. M. (1999). Fluid Mechanics, WCB (Boston, MA: WCB McGraw-Hill).

[B49] Wu C.-F. Akiyama S. (2002). Enhancement of damping performance of polymers by functional small molecules. Chin. J. Polym. Sci. 20, 119–127.

[B50] Yu H. Huang S. Chen G. Pan Y. Guo Z. (2015). Human–robot interaction control of rehabilitation robots with series elastic actuators. IEEE Trans. Rob. 31, 1089–1100. 10.1109/TRO.2015.2457314

